# Does Gratitude Ensure Workplace Happiness Among University Teachers? Examining the Role of Social and Psychological Capital and Spiritual Climate

**DOI:** 10.3389/fpsyg.2022.849412

**Published:** 2022-04-22

**Authors:** Naval Garg, Manju Mahipalan, Shobitha Poulose, John Burgess

**Affiliations:** ^1^University School of Management and Entrepreneurship, Delhi Technological University, Rohini, India; ^2^National Institute of Technology, Tiruchirappalli, India; ^3^Department of Management Studies, Torrens University Australia, Adelaide, SA, Australia

**Keywords:** gratitude, workplace happiness, spiritual climate, psychological capital, social capital

## Abstract

The study examines the necessity and sufficiency of gratitude for supporting workplace happiness among Indian university teachers. It also explores the mediating effect of psychological capital and social capital in the relationship between gratitude and workplace happiness. The moderating effect of spiritual climate is investigated. A survey of 726 university staff in India was undertaken to examine the relationship between gratitude and workplace happiness. A series of statistical tests involving correlation, multiple regression, and necessary condition analysis was undertaken from the data set. The mediation effect of psychological capital and social capital was investigated using bootstrapping estimates using PROCESS Macro in SPSS. Also, the moderation effect of spiritual climate was explored using PROCESS Macro in SPSS. The results reveal that gratitude is both a sufficient and necessary condition for workplace happiness. It also suggests a significant mediating effect of psychological capital and social capital. Also, a significant effect of spiritual climate amid the relationship between gratitude and workplace happiness is concluded. The study is one of the first studies that explore the relationship between gratitude and workplace happiness. It examines the mechanism through which gratitude influences happiness in the workplace.

## Introduction

Gratitude encompasses many ideas depending on its use and context. In simple terms, it can be expressed as a feeling of being thankful and appreciative. With gratitude, people cognize the goodness present in their lives which comes from sources within and out. Consequently, people tend to bond with something larger than themselves, for instance, other people, nature, or even higher power. The benefits of being grateful are numerous. Positive psychology research presents well-founded evidence to support the tangible relationship between wellbeing and gratitude (Emmons et al., 2019). Using Fredrickson’s (2004) broaden and build theory of positive emotions, researchers propose an upward spiral of gratitude and wellbeing where gratitude essentially creates an individual’s psychological, social and spiritual resources (Emmons and McCullough, 2003; Fredrickson, 2012). As research in the domain of gratitude is advancing, several underlying mechanisms through which gratitude supports long-term subjective wellbeing are also explored (Watkins, 2004). Apart from philosophical and psychological perspectives, recent research discusses the benefits of gratitude, especially for he32igher happiness levels, from a neuroscience standpoint. For instance, Ghosh (2018) recommends promoting a culture of gratitude in organizations as expressions of gratitude can release dopamine, the neurotransmitter that makes us feel good. From this vantage point, the present study looks at how gratitude could engender happiness in the workplace.

Workplace happiness is one of the most prized and sought-after goals for any organization. A happy workforce is a valuable asset, but ensuring sustainable happiness levels is a demanding task given the workplace’s potential uncertainties, multiple employee personalities, and variable leadership qualities. Previous researchers, including Emmons and McCullough (2003), Watkins et al. (2003), Park and Peterson (2006), Algoe (2012), and Nguyen and Gordon (2020), have reported a positive linkage between gratitude and happiness. The present study is novel in several ways. Firstly, it investigates the association between gratitude and happiness in the workplace. Previous studies are mainly confined to examining the general happiness level of the individuals. Secondly, the study examines both the necessity and sufficiency of gratitude for workplace happiness. To the best of our knowledge, no previous research has examined gratitude as a necessary condition for happiness. Thirdly, the relationship between gratitude and happiness has been explored through the lens of one’s psychological capital, social capital, and organizational spiritual climate current. Psychological capital (PsyCap) represents an individual’s positive psychological state characterized by the amalgamation of four constructs of self-efficacy, hope, optimism, and resilience (Luthans et al., 2007). PsyCap is empirically shown to be positively related to numerous work outcomes. Social capital indicates the network of relationships people form in the organization and the quality of such connections created (Adler and Kwon, 2002). Baker and Dutton (2017) put forward the idea of positive social capital for organizations to consider as they bolster individuals’ growth and thriving and flourishing. It is proposed that gratitude would result in greater PsyCap and social capital levels, which could be significant determinants of workplace happiness. The study, therefore, examines the mediating role of PsyCap and social capital. It suggests that the link between gratitude and workplace happiness could become more considerable by interacting with the organizational environment, which embodies some of the spiritual values relevant to the business. Accordingly, the moderating effect of organizational spiritual climate is also studied here.

The proposed study is theoretically premised on Fredrickson’s (2004) broaden-and-build theory of positive emotions. Positive emotions like gratitude “broaden people’s momentary thought-action repertoires and build their enduring social resources” (Fredrickson, 2004, p. 147). The author proposed two distinct processes- broadening of outlook and building of psychological and social resources. The first process, widening of thought-action tendencies, allows a grateful person to introspect and realize the importance of what one has in life. Also, it leads to a well-thought response instead of a narrow tit-for-tat reaction toward the benefactor. Gratitude broadens one’s outlook in three ways: Expanding the scope of attention, the range of cognition, and the scope of action (Fredrickson, 2003). The author further elaborates that this broadening process builds psychological capital for individuals. The feeling of abundance and appreciation for others fills one with hope, optimism, and resilience (Sarkar and Garg, 2021). Fredrickson also suggests that gratitude develops social capital in various ways. Firstly, the thoughtful exchange of benefits and gratefulness strengthens the bond between the benefactor and the beneficiary. Secondly, gratitude enriches one’s relationship with the divine, which helps one realize the universal oneness of consciousness (Watkins et al., 2015). Finally, gratitude expands one’s “more general skills for loving” (Fredrickson, 2004, p. 152). The virtue of loving allows individuals to establish a long-term social association with others. In this background, it is proposed that gratitude leads to workplace happiness through PsyCap and social capital development.

Bono et al. (2004) urged researchers to investigate various settings and populations which might benefit from the experience and expression of gratitude, including schools, and other educational settings. The authors pointed up the need for conducting cross-cultural studies as they are crucial in discerning the experiences of gratitude which are universal and diversified. Researchers have also called for more empirical research on gratitude and its concomitant benefits in modern work settings (Garg and Gera, 2019; Mahipalan and Sheena, 2019). In this background, the present study explores gratitude and workplace happiness among Indian university teachers. There is a renewed interest in exploring the happiness level of teachers for two fundamental reasons (Benevene et al., 2019). Firstly, teachers’ happiness has a direct bearing on their physical and psychological health and personal relationships. Secondly, since teachers are in direct and frequent contact with young minds, their happiness levels influence students’ intellectual and mental growth. Acknowledging the importance of happiness levels of teachers, the present study focuses on Indian university teachers. On that account, this study has, in essence, three objectives:

1. To review the gratitude-workplace happiness linkage in an educational context.
2. To examine the mediating effects of PsyCap and social capital in gratitude-workplace happiness relation.
3. To analyze the moderating effect of the spiritual climate.

## Theoretical Framework

### Gratitude and Workplace Happiness

Gratitude has been theorized from different viewpoints that entail an emotion, an attitude, an individual trait, and a coping strategy (Emmons and McCullough, 2003; Wood et al., 2010; Garg and Gera, 2019). According to Wood et al. (2010), gratitude is a life orientation directed toward identifying and valuing positivity. Considering this view, individuals may feel grateful for being alive, witnessing worthy life events, or simply being thankful for one’s abilities (Wood et al., 2010). However, even if a range of life events can generate feelings of gratitude, theoretically, gratitude can be generated from the experience of a constructive individual outcome that mainly depends on the acts of other people (Emmons and McCullough, 2003). Moreover, to understand the typical features of gratitude, one should study the characteristics of grateful individuals. Accordingly, Watkins et al. (2003) proposed four characteristics of grateful individuals. Firstly, grateful persons must have a sense of abundance; therefore, they do not feel deprived in life. Secondly, grateful persons would be thankful for the endeavor of others to contribute to their wellbeing. Thirdly, grateful persons would also be portrayed by the disposition to appreciate simple pleasures in their daily lives that are immediately available. Finally, grateful people should acknowledge the value of experiencing and conveying gratitude. In line with this, preceding studies have also identified the multidimensional aspect of gratitude. By considering the characteristics of grateful people, the present study also employs the Gratitude Resentment and Appreciation Test (GRAT) by Watkins et al. (2003), which consists of three dimensions: The sense of abundance, appreciation for simple pleasure, and appreciation to others for their worthy contribution to one’s life.

The present study focuses on how gratitude influences workplace happiness to deal with the inconsistencies in former findings (Watkins et al., 2003; Fisher, 2010; Nguyen and Gordon, 2020). The construct happiness at work and the related positive aspects of individuals focusing on happiness have received attention among practitioners for the past two decades. Over time, the construct has undergone conceptual modifications from its psychological and philosophical perspectives (Diener et al., 1999). Happiness is expressed as universal feelings of one’s life, satisfaction with one’s own life, the existence of constructive attitudes, and emotions with minimal levels of negative affect (Salas-Vallina et al., 2018). Nevertheless, past literature shows that it is hard to define or title particular emotions rather than positive attitudes at work. Previously, researchers have explored various concepts that largely overlap with the general term happiness. These include numerous positive attitudinal concepts: work satisfaction, commitment, employee wellbeing, work engagement, hedonic, and eudaimonia (Fisher, 2010). Though these concepts share certain similarities, each construct has its distinct meaning to address the broader aspects of positive attitudinal ideas. Overall, the construct of happiness at work broadly strives to demonstrate the theme of happiness at work compared to the majority of the attitudinal concepts, which are not adequate to elucidate the positive experiences at work (Salas-Vallina et al., 2018). According to Fisher (2010), elements of happiness have been postulated and measured at diverse levels. These levels include transient-level (happiness varies within the individual at different loci), person-level (happiness varies between individuals), and unit-level (happiness of teams, work units, or organizations as a whole) (Fisher, 2010). Happiness also has beneficial outcomes for both organizations and individuals at all levels. However, organizational research studies have been largely conceptualized at the personal level where all the variation of emotions occurs between individuals (Lyubomirsky and Lepper, 1999; Fisher, 2010). Therefore, the present study defined and measured happiness at the personal level.

Previous research has demonstrated the association between gratitude and happiness (Fisher, 2010; Nguyen and Gordon, 2020). For example, studies have recommended that individual positive qualities and gratitude facilitate true happiness (Fisher, 2010). People become happier and resilient when they express thanks to others because it strengthens relations, improves health, and lowers stress (Kausar, 2018). Along these lines, fostering gratitude may boost happiness while also creating positive attitudes at work. Although few studies have directly examined the longitudinal effects of dispositional gratitude on improved happiness at the workplace, the successful application of social cognitive theory and preceding studies with positive outcomes of inspiring people to express gratitude calls for excellent possibilities for happiness interventions (Emmons and McCullough, 2003). According to Ortony et al. (1988), gratitude is a blend of respect and pleasure experienced by people while taking a gift from the other person. Although these studies align with the theory that gratitude improves happiness, Watkins et al. (2003) suggested that gratitude could be an epiphenomenon of happiness. As noted above, individuals often attribute their happiness to external factors, thereby establishing a relationship between happiness and gratitude (Di Fabio et al., 2017).

According to Polak and McCullough (2006), grateful individuals exhibit greater happiness, more positiveness, and better life satisfaction with reduced depression and anxiety than less grateful individuals. Watkins et al. (2003) proposed improving subjective wellbeing while studying the expression and experience of gratitude, where people with more gratitude would feel happier than less grateful people (Watkins et al., 2003). Lately, Tachon et al. (2021) suggested that self-gratitude is associated with other important self-concepts such as self-acceptance and self-kindness, which are closely linked to individual wellbeing (Chamberlain and Haaga, 2001; Campos et al., 2016; Xu et al., 2016). Thus it can be construed that gratitude consistently establishes its relationship with greater happiness as it widens positive emotions and helps individuals experience pleasure from positive outcomes based on which we propose and test the following hypothesis:

**Hypothesis 1:** Gratitude will be positively related to happiness at the workplace.

### The Mediating Role of PsyCap

In positive psychology, psychological capital (PsyCap) is a notable contribution by Luthans et al.’s (2007) to depict the psychological capacities of individuals that can be developed, measured, and regulated for performance improvement. PsyCap reflects personal resources in simpler terms, addressing who you are as a person and what you can become (Avey et al., 2010). PsyCap is a multidimensional concept. It is defined as “an individual’s positive psychological state of development and is characterized by: (1) Having the confidence to take on and put in the necessary effort to succeed at challenging asks (self-efficacy) (2) making a positive attribution about succeeding now and in the future (optimism) (3) persevering toward goals and, when necessary, redirecting paths to goals to succeed (hope) and (4) when beset by problems and adversity, sustaining and bouncing back and even beyond to attain success (resilience)” (Luthans et al., 2007, p. 3). In general, the definition reveals PsyCap as a positive construct that integrates four positive individual psychological capacities, namely self-efficacy, hope, optimism, and resilience, ensuring positive strength and performance improvement. It is evolving as a significant path to recognize, appreciate, and foster ideal employee performance in the workplace (Rehman et al., 2017). Preceding studies have established the benefits of PsyCapboth at the individual and organization levels. While the individual level accounts for a psychological capacity that enhances individuals’ growth and performances, the organizational level considers return on investment, leverage, and competitive advantage to improve employee performance (Sarkar and Garg, 2020). Moreover, it is deemed relevant in developing human resources because it helps managers develop, measure, and leverage the efficiency of their employees to use as a competitive advantage attaining organizational success (Ardichvili, 2011).

Past studies have revealed a positive link between PsyCap and employee behaviors and attitudes (Luthans et al., 2007). Gratitude entails an emotion, an attitude, and an individual trait (Emmons and McCullough, 2003; Garg and Gera, 2019). Studies also hinted that individuals who routinely go through positive emotions through dispositional gratitude might also be likely to acquire PsyCap (Luthans et al., 2007). Furthermore, the relationship between gratitude and PsyCap arises from an individual’s capacity to effectively use one’s psychological resources (i.e., hope, efficacy, resilience, and optimism), resulting in positive outcomes in the work domain (Ahrens, 2016). Mccullough et al. (2002) proposed that the dimensions of PsyCap, such as hope and optimism, are positively correlated with gratitude. Along these lines, gratifying life responses can lead to happiness, peace of mind, psychological health, and, more significantly, fulfilling personal relationships (Emmons and McCullough, 2003). Prior research reported a positive association between PsyCap and subjective wellbeing. Studies in PsyCaphave offered a meaningful outcome on employee subjective wellbeing resulting in workplace happiness (Avey et al., 2010; Khandelwal and Khanum, 2017). Two related happiness constructs—workplace happiness and subjective wellbeing emphasize hedonic aspects of wellbeing that maximize an individual’s positive emotions and pleasure (Fisher, 2010). Even though both concepts signify personal perceptions of wellbeing and satisfaction, they can differ based on the domain in which people experience happiness. Choi and Lee (2014) demonstrated that PsyCap is positively related to subjective wellbeing and perceived workplace happiness among South Korean employees.

Additionally, precedent studies have shown a positive relationship between PsyCap and workplace wellbeing with improved job satisfaction. Evidence suggests that employees with improved PsyCapare more likely satisfied with their work and organization (Khandelwal and Khanum, 2017) and may avoid early career burnout and ensuing personal and work-related consequences (Spence Laschinger and Fida, 2014). While examining the predictors of happiness at the workplace, Joo and Lee (2017) found employees with improved PsyCapthat include self-efficacy, hope, resilience, and optimism are more likely to be satisfied with their work resulting in better life satisfaction. Based on the above discussion, the following hypothesis is formulated to establish the relationship between gratitude, PsyCap, and workplace happiness.

*H2a: PsyCap mediates the relationship between gratitude and workplace happiness.*

### The Mediating Role of Social Capital

Social capital is defined as “the sum of the resources, actual or virtual, that accrue to an individual or a group by virtue of possessing a durable network of more or less institutionalized relationships of mutual acquaintance and recognition” (Bourdieu and Wacquant, 1992, p. 14). In simple words, social capital encompasses the resources concerning “whom you know” (Luthans and Youssef, 2004). These resources are social relationships, networks, communities, societies, and the flow of information that yield the development and growth of human capital (Coleman, 1988; Machalek and Martin, 2015). Understandably, social capital is a form of capital that can generate profits or returns like any other physical capital (machinery, building) and human capital (skills and personality attributes) (Melé, 2003). For instance, a secure and stable family background can reinforce academic success and support the growth of highly respected skills and qualifications. Social capital includes all forms of individual resources that evolve from social relationships, social gatherings, and social support (Choi and Dinitto, 2013). Researchers predominantly use two forms of social capital known as bonding and bridging (Woolcock, 1998). Bonding social capital signifies the link with others broadly homogeneous in nature, whereas bridging social capital implies linking a community with different sets of people in various degrees remarkably heterogeneous group people (Melé, 2003). Besides these forms of social capital, individuals can better recognize the influence of social capital if there is a clear-cut emphasis on the interaction between themselves and how this interaction varies over time. It is worth noting that people can possess bonding social capital without bridging social capital but not vice versa (Melé, 2003).

According to Slåtten et al. (2019), investing in social capital helps organizations work more effectively and influences employee behaviors, attitudes, norms, and work performance. Moreover, grateful individuals approach others to widen the social network as they consider others valuable (Mpinganjira, 2019). The greater the degree an individual can depend on their social structures and network, the better their social capital. Henceforth grateful individuals tend to behave and work more ethically and prosocially (Garg et al., 2021). People experiencing gratitude are more likely to repeatedly help out their beneficiaries, ensuring gratuitous deeds are reinforced (Watkins et al., 2003). Recent literature also emphasizes the importance of social capital in elucidating workplace happiness (Majeed and Samreen, 2021) and demonstrates social capital as a principal source of happiness at work. Borooah (2006) commended the importance of the social network, social support, and family (intangible capital) for individuals. This intangible capital plays a pivotal role in settling conflict at the workplace, resulting in increased workplace happiness. Past evidence also shows that people with lower social capital are likely to experience less happiness at work (Majeed and Samreen, 2021). Based on the above discussions, people with more gratitude are likely to experience better Social capital, resulting in improved happiness at the workplace. Thus the study hypothesizes that:

*H2b: Social capital mediates the relationship between gratitude and workplace happiness.*

### The Moderating Role of Spiritual Climate

Work climate is about employee perceptions of the organizational environment. These perceptions have significant personal meaning for individuals as these environmental aspects are interpreted in terms of individuals’ values (Yoon et al., 2001). Such perceptions are important as they impact the behavioral and attitudinal responses of the individual more than the environment itself (Chirico, 2016). In the context of workplace spirituality, where researchers try to understand how spirituality unfolds and manifests for employees at workplaces, work climate could act as a mechanism to integrate an individual’s spirituality with their work. Pandey et al. (2009) had conceptualized spiritual climate using the dimensions of swadharma, lokasangraha, sense of community, and authenticity. The concept of swadharma finds its place in numerous Indian epics and spiritual texts and is most illuminatingly explained in the Shrimad Bhagwat Gita. Gita states Swadharma as the righteous conduct of self-based on the identification of one’s ability. Since Swadharma has been closely related to an individual’s state of being, it has been defined as meaningful and meditative work. Meaningful work is work that is perceived to be purposeful and substantive by the individual (Ashmos and Duchon, 2000). Meditative work is absorbing and engaging. It allows individuals to be engrossed in the job they perform. The expression lokasangraha is also found in Bhagavad Gita, which presents the notion of working toward all welfare. The true calling of a person lies in lokasangraha, where they transcend the egocentric motives by seeing the self as part of everything (Pandey et al., 2009). Sense of community refers to the interconnectedness and interdependence among people (Giacalone and Jurkiewicz, 2010). It indicates a sense of belonging, mattering, and a shared emotional connection with one another (McMillan and Chavis, 1986). Authenticity is when the external behavior of people is in alignment with their internalized values and beliefs (Pandey et al., 2009). Lehman et al. (2019) also support the congruence between an entity’s internal values and external expressions as one of the core dimensions of authenticity. Chirico et al. (2020) reported the spirituality reduces stress and anxiety among teachers. Similarly, Chirico (2017) concluded healthy relationship between spirituality of Italian teachers and their mental health.

In addition to developing and validating spiritual climate inventory, Pandey et al. (2009) demonstrated the positive impact of spiritual climate on employees’ service performance. Although the study of spiritual climate has been minimal in organizational contexts, researchers have shown significant findings that can be adduced to explain the potentiality of spiritual climate to promote spirituality at a collective level. For instance, the spiritual climate positively affected team learning and resulted in team innovative behavior (Pandey et al., 2016, 2019). Using the social cognitive theory framework, which underscores the relevance of contexts or environment in the individual’s knowledge acquisition and behavioral responses, Sarkar and Garg (2021) explain the role of individual spirituality combined with the spiritual climate of the organization in promoting non-violent workplace behavior.

The dimensions of spiritual climate highlighted here have consequential implications for workplace happiness. Meaningful work has been contemplated as a multifaceted eudemonic psychological state. Engaging in such work has been argued to be a fundamental human need contributing to greater levels of wellbeing (Yeoman, 2014; Bailey et al., 2019). Likewise, Dambrun and Ricard (2011) proposed that selfless psychological functioning promotes stable and authentic happiness in individuals to strengthen harmony with oneself and others. Self-transcendence allows people to surpass their self-interests and contribute toward a higher purpose with a favorable association with emotional regulation, perceived levels of relational quality, and life satisfaction, all of which are facets of subjective wellbeing (Le, 2011; Kao et al., 2016). There is a consensus on the positive relationship between a sense of community and wellbeing, mostly in various settings (Stewart and Townley, 2020). Davidson and Cotter (1991) found that a sense of community was significantly related to subjective wellbeing facets and was most robust for the happiness dimension. Researchers have consistently demonstrated a sense of community as a substantial predictor of employee wellbeing in public service organizations. Authenticity has generally been a vital explanatory variable for subjective wellbeing as authentic people can act in alignment with their interests and values, thus reducing psychological conflicts (Kifer et al., 2013; Akin and Akin, 2014). Zheng et al. (2020) reported that authenticity leads to higher levels of mindfulness, resulting in elevated levels of individual happiness. Based on the above literature, it is plausible that gratitude combined with spiritual climate can upraise workplace happiness and following hypothesis is postulated:

*H3: Spiritual climate moderates the relationship between gratitude and workplace happiness.*

## Methodological Framework

### Objective and Data Collection

The primary objective of the study was to explore the sufficiency and necessity of gratitude for contributing to the happiness among Indian teachers. The study also examined the mediating effect of social capital and psychological capital in the relationship between gratitude and workplace happiness. The data was collected from 726 teachers engaged in different public and private universities situated in the Delhi-National Capital Region with the help of multistage random sampling. Initially, fifteen institutes, each in public, and private categories, were randomly chosen. Then, five departments were randomly selected in these thirty chosen institutes. Questionnaires were sent to ten faculty members in each 150 chosen departments. Thus, initially, 1,500 respondents were approached. Podsakoff et al. (2012) suggested that data were collected by ensuring temporal and psychological separation to avoid common method bias. Responses related to the independent variable (gratitude), mediators (social and psychological capital), and dependent variable (workplace happiness) were collected in a time gap of 2 months each. Firstly, a gratitude questionnaire was sent to 1,500 teachers through emails, out of which 1,272 responded. Secondly, social and psychological capital questionnaires were sent to these 1,272 teachers after a gap of 2 months. One thousand fifty-nine responses were received in the second round of data collection. And lastly, happiness questionnaires were emailed to these 1,059 teachers again after a gap of 2 months, out of which 779 replied. Fifty-three responses were discarded for being incomplete. Also, different cover stories/backgrounds were given to each data collection stage. Such psychological separation reduced common method bias by making respondents believe that studies are unrelated (Podsakoff et al., 2012).

The sample consisted of 400 female and 326 male faculty members. Also, 475 respondents were from government institutes, and the rest (251 respondents) were from private educational institutes. Also, respondents belonged to different age categories, i.e., up to 30 years (24%), 31–40 years (42%), 41–50 years (20%), and above 50 years (14%). The average experience of employees in the current organization was 6.8 years, and the total average experience was 14.8 years. Nearly 78% of participants were permanent employees, and the remaining 22% were contractual teachers. Also, 539 respondents were married, and the remaining 187 were unmarried. Since the sample was heterogeneous, the effect of sample characteristics (age, gender, marital status, and nature of employment) on gratitude (independent variable) and workplace happiness (dependent variable) was examined using *t*-test or ANOVA. All these sample characteristics-based differences were statistically insignificant at a 0.05 level of significance. Table 1 illustrates the demographic description of the sample.

**TABLE 1:** Demographic description of the sample.

Variables	Category	Percentage
Gender	Male	55%
	Female	45%
Age	Up to 30 years	24%
	31–40 years	42%
	41–50 years	20%
	Above 50 years	14%
Marital status	Married	74%
	Unmarried	26%
Employment	Permanent	78%
	Temporary	22%

*Primary data.*

### Measures

#### Gratitude

Gratitude was measured using Watkins et al.’s (2003) GRAT-16 (short form) that accessed three dimensions of gratitude, namely a lack of sense of deprivation (6 statements), simple Appreciation (6 statements), and appreciation for others (4 statements). These statements were rated on a nine-point scale ranging from 1 (strongly disagree) to 9 (strongly agree). The questionnaire included statements like- “I couldn’t have gotten where I am today without the help of many people,” “Life has been good to me.”

#### Psychological Capital

Luthans et al.’s (2007) PsyCap scale was used to access the psychological capital of respondents. The scale measured four dimensions of psychological capital- self-efficacy (3 statements), hope (4 statements), resilience (3 statements), and optimism (2 statements) on a six-point rating scale ranging from strongly disagree (1), disagree (2), somewhat disagree (3), somewhat agree (4), agree (5) and strongly agree (6). Examples of statements are—“I feel confident contributing to discussions about the company’s strategy,” “I can get through difficult times at work because I have experienced difficulty before,” and “I am optimistic about what will happen to me in the future as it pertains to work.”

#### Social Capital

Social capital was measured using the Personal Social Capital Scale-16 developed and validated by Wang et al. (2014). It was a two-factor scale comprising bonding capital and bridging capital subscales rated on a five-point rating scale. Items included in the scale were “How do you rate the number of your country fellows/old classmates?” “Among your coworkers/fellows, how many you can trust?” and “Among your relatives, how many you can trust.”

#### Workplace Happiness

Lyubomirsky and Lepper’s (1999) four-item Subjective Happiness Scale was used to define an individual’s happiness level. This questionnaire was slightly modified to explore happiness levels at the workplace. One of the original statements was, “Compared to most of my peers, I consider myself: less happy vs. more happy.” It was modified as, “Compared to most of my office colleagues, I consider myself: less happy vs. more happy.” Similarly, another statement, Some people are generally very happy. They enjoy life regardless of what is going on, getting the most out of everything. “To what extent does this characterization describe you?” was modified as Some employees are generally very happy. They enjoy life regardless of what is going on, getting the most out of everything. “To what extent does this characterization describe you?.” It was a seven-point rating scale ranging from 1 (less happy) to 7 (more happy).

### Reliability and Validity

Reliability was defined as how consistent results are obtained under similar conditions (Kumar et al., 2018). Internal consistency reliability was measured using Cronbach’s alpha, which represented an average of all possible split-half correlations for a set of items. The value of Cronbach’s alpha should be greater than 0.70 (George and Mallery, 2003). The Cronbach’s alpha values for variables of the study were- a gratitude = 0.804, social capital = 0.768, psychological capital = 0.887, workplace happiness = 0.839. Cronbach and Shavelson (2004) suggested the calculation of composite reliability along with Cronbach’s alpha values. Again, all Composite reliability estimates were reported to be greater than 0.70, confirming reliability (Hair et al., 2010). Average Variance Explained (AVE) values were calculated to access convergent and divergent validity. AVE must be greater than 0.50 for convergent validity (Fornell and Larcker, 1981). The AVE values for variables of the study were- gratitude = 0.719, social capital = 0.664, psychological capital = 0.791, workplace happiness = 0.626, and spiritual climate = 0.731. These values reflect acceptable psychometric properties (reliability and validity) of different measures, i.e., gratitude, psychological capital, social capital, workplace happiness, and spiritual climate. Although these scales were developed in the western context, the values of Cronbach’s alpha, composite reliability and AVE recommend their suitability in the Indian context too.

### Common Method Bias

As suggested by Podsakoff et al. (2012), several procedural and statistical procedures were adopted to minimize the effect of common method bias. Firstly, the study used questionnaires with different rating scales, gratitude (nine-point scale), psychological capital (six-point scale), social capital (five-point scale), and workplace happiness (four-point scale). It helped eliminate method bias resulting from common scale properties (Podsakoff et al., 2012). Secondly, survey participants were ensured of confidentiality and strict academic use of collected data. It secured a more accurate and positive response from respondents (Podsakoff et al., 2007). Thirdly, temporal and psychological separation of independent and dependent variables were maintained. Such detachments reduced the salience of the linkage between the predictor and the criterion variable, which reduces common method bias (Podsakoff et al., 2012). Also, common method variance was examined using the Harman one-factor test. When factor analysis using varimax rotation was applied on all items of the study, no single factor was reported that explained the majority of the variance. The maximum variance explained by the most significant factor was 28.31%; thus, common method bias was insignificant in the present study (Chang et al., 2010).

### Analysis of Collected Data

The mean and standard deviation of all variables (gratitude, psychological and social capital, and workplace happiness) were calculated to examine the status of gratitude and workplace happiness among faculty members. Further, the relationship between gratitude and workplace happiness was explored with the help of correlation, multiple regression, and necessary condition analysis using SPSS and R-studio. The mediation effect of psychological capital and social capital was investigated using bootstrapping estimates using Hayes and Scharkow’s (2013) PROCESS Macro in SPSS. Previous researchers like Hayes and Scharkow’s (2013) and Hayes’s (2017) argued that PROCESS provided better results than the traditional Baron and Kenny’s (1986) mediational model. Also, Shrout and Bolger (2002) suggested that the bootstrap method outperformed other mediation methods in several ways. The bootstrap method could be applied for non-normal/skewed data. The technique proved to be equally powerful for binary or ordinal mediating variables. Also, it provided effective results for small samples too. Finally, the moderating effect of the spiritual climate was also accessed using Hayes and Scharkow’s (2013) PROCESS Macro in SPSS.

## Results

Table 2 illustrates descriptive statistics and a correlation matrix of all study variables. Gratitude was positively correlated with psychological capital and social capital. These findings were in line with the proposition of Fredrickson’s broaden-and-build theory. Also, psychological capital and social capital reported a significant positive correlation with workplace happiness. In other words, the finding suggests that resourcefulness (psychological and social) makes an employee happier at the workplace. In addition, spiritual climate of an organization was also found positively associated with gratitude (*r* = 0.254, *p* < 0.01), psychological capital (*r* = 0.212, *p* < 0.01), social capital (*r* = 0.350, *p* < 0.01), workplace happiness (*r* = 0.341, *p* < 0.01). Table 3 reported that three gratitude significantly predicts workplace happiness (β = 0.472, *p* < 0.05). Results also suggested that three dimensions of gratitude explained 54.2% variations in workplace happiness (R-square value = 0.542, *p* < 0.05). It indicates the acceptance of H1, i.e., gratitude is positively related to workplace happiness. Also, the following regression equation was derived.

**TABLE 2:** Descriptive statistics and correlation matrix.

Variable	Mean	S. D	CA	CR	AVE	G	PC	SC	WH	S
G	7.01	1.124	0.804	0.923	0.719	1				
PC	4.24	0.689	0.887	0.912	0.791	0.284*	1			
SC	3.86	0.693	0.768	0.843	0.664	0.259*	0.301*	1		
WH	3.06	0.851	0.839	0.819	0.626	0.298*	0.163*	0.283*	1	
S	3.26	0.628	0.811	0.944	0.731	0.254*	0.212*	0.350*	0.341*	1

Primary data. *Significant at 0.01, CA, Cronbach’s alpha; CR, Composite reliability; AVE, Average Variance Explained; G, Gratitude; PC, Psychological Capital; SC, Social Capital; WH, Workplace Happiness; S, Spiritual Climate.

**TABLE 3:** Results of the regression equation.

Variables	Unstandardized coefficients		Standardized coefficients	*t*-value	Sig.	R-square	*F*-value	Sig
	B	Std. Error	Beta					
Constant	1.688	0.270		6.241	0.000*	0.542	26.54	0.00*
G	0.431	0.032	0.472	2.620	0.009*			

Primary data. *Significant at 0.05, G, Gratitude.

WP=1.688+0.472⁢G

Dul (2016) argued that regression examines only sufficiency and not the necessity of the condition. The above equations only reported the sufficiency of gratitude for workplace happiness. It was argued that regression is premised on additive causality, i.e., Y = a + b_1_X_1_ + b_2_X_2_ + b_3_X_3_+. The reduction in the value of any predictor variable resulted in a decline in the value of the criterion variable. However, such a decrease of the dependent variable may be compensated by increasing any other independent variable (Dul, 2016). In a nutshell, regression analysis assumed that independent variables are sufficient to improve the outcome. Necessary Condition Analysis examines the necessity of a condition. “A necessary cause is a constraint, a barrier, an obstacle, a bottleneck that must be managed to allow the desired outcome to exist. Every single necessary cause must be in place, as there is no additive causality that can compensate for the absence of the necessary cause” (Dul, 2016, p. 11). Dul (2016) suggested that the necessary condition is based on multiplicative causality (i.e., Y = a. b_1_X_1_. b_2_X_2_. b_3_X_3_ …). And, thus, the zero value of any independent variable may lead to zero outcomes.

In summation, the occurrence of necessary conditions is mandatory for the outcome event to happen; however, the fulfillment of necessary requirements does not guarantee the outcome variable’s presence (Dul, 2016). Dul (2016) proposed a simple mechanism to investigate the necessity of an independent variable using R-studio. The author suggested that necessary conditions could be explored by plotting a scatter diagram taking the independent variable on *X*-axis and the dependent variable on *Y*-axis. A ceiling line segregates an area without observations (“Empty Zone”) from an area with observations (“Full Zone”) is drawn. And, the presence of an empty upper-left corner region of the graph confirms the necessity of the condition (Dul, 2016). It was also proposed that the effect size would indicate the strength of the necessary condition. Value of effect size lies between zero and one such that-

`⁢`⁢0<d<0.1-small⁢effect,

0.1≤d<0.3-medium⁢effect,

0.3≤d<0.5-large⁢effect,and

$$0.5\leq d<1.0-\mathrm{very}\mathrm{large}\mathrm{effect}.^{\prime \prime }$$

**FIGURE 1:**
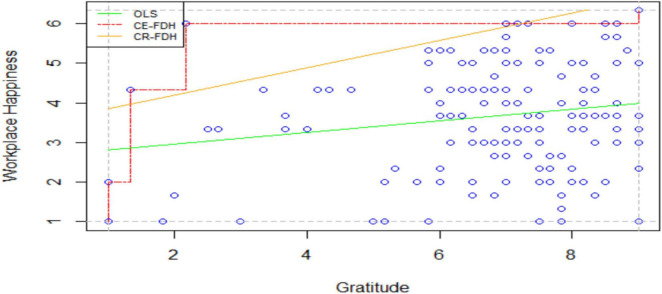
NCA plot (gratitude and workplace happiness). Primary data.

It was also suggested to use correlation, regression, and necessary condition analysis simultaneously to have a deep insight into the two variables’ causal relationship. Here, Figure 1 investigated the necessity of gratitude for workplace happiness. An empty upper-left corner region revealed that gratitude is a necessary condition for workplace happiness. Gratitude has a “medium necessary effect” (0.10 < *d* < 0.30) on workplace happiness (refer to Table 4).

**TABLE 4:** Effect size of necessary condition.

S. No	Plot	Effect size		Interpretation
		CE-FDE	CR-FDH	
1	G Vs. WH	0.107	0.082	Small effect

*Primary data. G, Gratitude; WH, Workplace Happiness.*

As proposed by the broaden-and-build theory of gratitude, the parallel multiple mediation effect of psychological and social capital was examined with PROCESS macro in SPSS. Table 5 confirmed a significant mediation effect of psychological and social capital amid the relationship of gratitude and workplace happiness. The indirect effect size was 0.3159, and zero did not lie between LLCI and ULCI. It was interesting to acknowledge that social capital was a more potent mediator (effect size = 0.2116) than psychological capital (effect size = 0.1043). Also, the direct effect of gratitude on workplace happiness (effect size = 0.0242) was much lesser than the indirect effect (effect size = 0.3150).

**TABLE 5:** Results of parallel multiple mediation.

Variable	Effect	SE	LLCI	ULCI
**The total effect of gratitude on workplace happiness**				
Gratitude	0.3401	0.0406	0.2604	0.4199
**Direct effect of gratitude on workplace happiness**				
Gratitude	0.0242	0.0430	0.2314	0.4004
**Indirect effect of gratitude on workplace happiness**				
Total	0.3159	0.0150	0.0052	0.0543
Psychological capital	0.1043	0.0169	0.0037	0.0574
Social capital	0.2116	0.0196	0.0063	0.0145

*Primary data.*

Table 6 illustrates the moderating effect of spiritual climate in the relationship of gratitude on workplace happiness. Since there are different models available in PROCESS macro, model 1 was selected to study the moderation effect. The results highlighted a significant interaction effect (*t*-value = 1.260, *p* < 0.05), and thus moderation effect of spiritual climate is concluded. Table 7 indicated that the moderation effect of spiritual climate is 8.41% (*R*^2^ change = 0.0841, *p* < 0.05).

**TABLE 6:** Results of moderation analysis.

Model	Coefficient	S.E	*t*-value	P	LLCI	ULCI
Constant	3.064	0.442	13.892	0.000*	1.283	2.849
G	2.340	0.329	4.042	0.002*	2.540	2.875
SC	1.932	0.225	4.743	0.000*	0.554	0.849
Interaction (G*SC)	3.132	0.830	1.260	0.042*	2.949	3.940

Primary data. G, Gratitude; SC, Spiritual Climate. *Significant at 0.05.

**TABLE 7:** Test of higher-order unconditional interactions.

Model	R^2^ change	*F*-value	*p*
Interaction (G*SC)	0.0841	12.112	0.030*

Primary data. G, Gratitude; SC, Spiritual Climate. *Significant at 0.05.

## Discussion

The study fundamentally revolves around the question of whether gratitude elevates workplace happiness. This connection between gratitude and happiness has briefly been hinted upon in a handful of studies. For instance, researchers have suggested that practicing character virtues, including gratitude, could facilitate authentic happiness (Seligman et al., 2005; Lyubomirsky, 2008; Fisher, 2010). It is also worthwhile to note that happiness researchers have used the term happiness and subjective wellbeing interchangeably ascribable to the complexity of a psychological construct which is significant but remains untellable to a large extent (Lyubomirsky and Lepper, 1999; Lyubomirsky, 2001; Seligman, 2002; Joo and Lee, 2017). Polak and McCullough (2006) presented gratitude as an antidote for materialism and called for empirical research to demonstrate the causal relationship between gratitude and happiness. Alkozei et al. (2018), while observing the lack of studies research on the differential contribution of gratitude on subjective wellbeing, proposed cognitive and psycho-social frameworks to demonstrate how gratitude may lead to wellbeing. The results of the first causal model depicting the direct effect of gratitude on happiness could be reviewed in light of these frameworks.

The cognitive framework outlines gratitude as a cognitive-emotional process that results in positive attentional bias, positive interpretation bias, and positive memory bias, enhancing wellbeing. A feeling of abundance and the ability to appreciate simple pleasures in life could affect a person’s cognition to see negative or ambiguous situations in a positive way and focus more on positive stimuli within the environment. This finding corroborates with the findings of Simons et al. (2020), who revealed the affective pathway between a sense of abundance and positive affect. Positive affect, owing to its adaptive value, broadens the mindsets and brings in behavioral flexibility in people, unlike negative emotions and the personal resources accrued during these moments of positivity are durable (Fredrickson and Losada, 2005; Rowe et al., 2007; Fredrickson, 2013). The third dimension of gratitude which is the appreciation for others, might explain how gratitude leads to wellbeing through psycho-social mechanisms. When people acknowledge and express their gratitude, the quality of interpersonal relationships is augmented as it brings in an opportunity for closer human connections (Algoe, 2012).

Furthermore, the behavioral expression of gratitude results in more social support offered and received by grateful individuals, which ultimately leads to higher levels of wellbeing. Alongside these findings, another possibility is that of gratitude magnifying wellbeing through the act of savoring through which people derive happiness from positive events (Jose et al., 2012; Szczygieł and Mikolajczak, 2017). Savoring, where constant mental repetition of a positive experience happens, could assist the upregulation of emotions and increase attention on positive instances from the past.

The next part of this discussion dwells on the results of the parallel mediational effects of psychological and social capital on the gratitude-happiness relationship. The results signal that both psychological and social capital are potential mediators in the gratitude-happiness relationship. The evidence of psychological capital as a mediator is in congruence with the findings from research where PsyCap was modeled as a predictor of workplace happiness (Culbertson et al., 2010; Choi and Lee, 2014; Williams et al., 2015). It reinforces the earlier propositions that positive capacities such as resiliency, hope, optimism, and self-efficacy beliefs are instrumental in promoting overall happiness in life. Youssef-Morgan and Luthans (2015) proposed cognitive, affective, conative, and social mechanisms triggered by PsyCap, leading to happiness and wellbeing. For instance, PsyCap encourages positive appraisal and interpretation of situations which could boost individual perseverance, motivation, and efforts. Different components of PsyCap could create a varied range of positive states, which can broaden an individual’s thought-action repertoires. Thirdly, PsyCap capabilities can lead the individual to build effective, realistic goals, intentional actions to pursue these goals with a sense of control. Lastly, PsyCap can activate the social mechanism through increased interaction, high-quality relationships and networks, and improved communication. PsyCap might also be efficacious in abating the negativity bias to a certain extend by dint of these mechanisms. It is critical to keep a rein on negative emotions as they have broader repercussions on the self than positive emotions (Baumeister et al., 2001).

Social capital is another mediator that has been examined. The findings pertaining to the association between social capital and wellbeing confirm the conclusions from the limited number of studies carried out (Helliwell and Huang, 2010; Han et al., 2013; Klein, 2013; Han, 2015; Yamaoka and Yoshino, 2015; Munzel et al., 2018; Tsuruta et al., 2019; Berraies et al., 2020). It may be noted that barring a few exceptions, most of these studies were conducted in a non-workplace context. The effect of social capital on happiness can be construed using some suggested mechanisms. Firstly, trust is an important element in social relationships, and trust has been positively related to the wellbeing of individuals. People who place a higher level of trust in their social relationships might experience lesser levels of sadness and loneliness which naturally benefit their overall wellbeing. Although the relationship between trust and happiness remains empirically underexplored, studies support this postulation (Bjørnskov, 2003; Portela et al., 2013; Hu, 2020). Social capital is characterized by a sense of connectedness that can facilitate the psycho-social process, including self-esteem, respect for each other, and social support, all of which can lead to individual wellbeing. Social capital could also be instrumental in improving the quality of work-life through more open and constructive social interactions where employees feel valued and appreciated. When the quality of work-life is enriched, that could gradually raise the happiness levels. Finally, social capital can promote prosocial behaviors and altruistic emotions, which are significantly related to life satisfaction and wellbeing. Since social capital was found to be a more influential mediator than psychological capital, we may deduce that a combination of these mechanisms was at play in the relationship between gratitude and happiness.

The focus now shifts to the moderation effects of spiritual climate on the relationship between gratitude and workplace happiness. The spiritual climate was found to positively influence the gratitude-happiness connection, suggesting that instances of gratitude engendering happiness are more probable when the organization climate is spiritually conducive. These findings may seem intuitive, especially when we look at the operationalization of spiritual climate in this study. The four dimensions of spiritual climate, namely, swadharma, sense of community, lokasangraha, and authenticity, empower an individual to engage in meaningful work in alignment with one’s inherent nature, soak up in the feeling of belongingness, put in efforts for the common good and to act genuinely and openly consistent to his/her core internalized values and beliefs. When people bring more passion and purpose to work, which originally stems from their core values and principles in life, it can act as a critical contributor to happiness. Meaning is the attraction for many individuals, and a positive spiritual climate bolsters meaningfulness at work (Achor et al., 2018; Wu et al., 2020).

Sutton’s (2020) meta-analytic review concludes that evidence demonstrates the positive relationship between authenticity and wellbeing. Staying true to oneself stimulates one’s self-esteem and earns social support besides acting as a buffering mechanism against distress caused by anxiety and strain. Thirdly, volunteering for others’ benefits can increase empathic emotions in people who can contribute to happiness. It is particularly true in the case of eastern traditions where self-transcendence supersedes the concept of self-enhancement because the self is considered as a small part of the collective and cosmos (Joshanloo, 2014). A strong sense of connectedness can buoy social relations satisfying our inherent need to bond with each other. We have a relational self which is equally important as our individualized self, and tending to it could contribute to our happiness.

### Theoretical Implications

The present study proposes to enrich existing literature in many ways. Firstly, the study explores the utility of gratitude in the organizational context. Previous researchers, including Fredrickson (2004) and Watkins (2014), highlighted the scarcity of gratitude studies among working professionals. They encouraged future researchers to examine gratuitous work behavior to realize the true potential of gratitude practices and interventions. It is one of the few studies investigating the linkage between gratitude and workplace happiness. Secondly, researchers have acknowledged the relevance of happiness research in organizational settings. It contributes to the explication of several different phenomena such as job satisfaction, work performance, and motivation and reckons that happiness has significant applied consequences (Judge and Kammeyer-Mueller, 2017). Happiness as a positive, stable, and measurable construct has not been explored extensively, and literature on its antecedents and consequences is sparse (Thompson and Bruk-Lee, 2020). This study addresses this gap by capturing the distinct construct of employee happiness and looks at factors that enhance employee happiness. Thirdly, a few scholars have highlighted the positive association between gratitude and happiness (please refer, Watkins et al., 2003; Park and Peterson, 2006; Nguyen and Gordon, 2020). The present study augments these findings by examining the underlying mechanism through which gratitude influences the happiness of teachers. The results suggest the significant mediating effect of PsyCap and social capital. In simple words, the findings elaborate that gratitude increases one’s psychological and social capital, which triggers happiness in the workplace. Fourthly, the study provides an alternate description of the broaden-and-build theory of positive emotions. It could be inferred that gratitude broadens the psychological and social capitals of individuals. These expanded capitals build resources for happiness. This alternate explanation of theory is particularly crucial because this study is conducted in non-western settings. Previous gratitude studies are mostly confined to western contexts (Garg et al., 2021). Thus, the study’s findings are poised to increase the acceptability of the broaden-and-build theory in eastern countries. And lastly, the study also enriches the domain of workplace spirituality. Researchers like Giacalone and Jurkiewicz (2003), Sheep (2006), Gatling et al. (2016), and Pawar (2016) highlighted the importance of integrating spirituality and work. Working without the involvement of the employee’s spirit is tedious (Saxena et al., 2020). The current study examines the moderating effect of the organizational spiritual climate. The finding suggests that institutionalization of spiritual values at the workplace facilitates greater gratitude-induced workplace happiness. In other words, gratitude is a better antecedent of happiness in the workplace for spiritual organizations.

### Practical Implications

Arguably, teachers in higher education play a crucial role in building society and carry out a complex set of functions in the process. Teaching is one of the most revered jobs; however, it is equally demanding as it is affected by ongoing and unpredictable challenges. Studies have shown that teacher wellbeing has direct consequences on the emotional and academic outcomes of students. From this standpoint, the study carries several practical implications for educational institutions. Since gratitude has been identified as a potential predictor of workplace happiness, teachers can develop an attitude of gratefulness. Although considered primarily dispositional, research has revealed that interventions are constructive for developing gratitude. Researchers and practitioners advocate a wide range of gratitude interventions through actions such as a “gratitude journal,” “gratitude letter and subsequent visits,” “gratitude meditation,” “cultivating mindfulness,” “gratitude collages,” “sharing positive events,” “act of kindness,” and “progressive muscle relaxation techniques.” These interventions could support university teachers to develop gratefulness. University administration may support the development of a gratuitous workplace through organizing various events like “thanks-giving day,” “joy of giving week,” “self-introspection,” etc. Howells (2014) suggested that a grateful teacher is beneficial for students’ academics and career growth. She proposed a gratitude approach specifically for teachers. In this practice, teachers are encouraged to realize and examine their innermost gratuitous attitude. Innermost attitude is defined as “thinking from the depths of one’s being” or “spirit in which one does a certain action” (Howells, 2014, p. 3). They are also taught to utilize this realization in their teaching practice and learning outcomes. It is also suggested that gratitude is contagious. And a grateful teacher is more likely to imbibe feelings of thankfulness, abundance, and appreciation among students (Garg, 2020).

In a similar vein, organizations should create adequate opportunities for teachers to develop their psychological capabilities. This involves training teachers to continuously identify their values, strengths, and abilities and how to apply them skillfully to respond to uncertainties and evolving circumstances. Moreover, a dynamic skill development approach should be adopted to enable teachers to add value to their work steadily. Social relationships have extreme significance in educational settings, and when the dynamics are positive, the group works well together. Organizations should look at wellbeing as a collective outcome where individuals sense a share of belongingness and a high degree of situational happiness. Equally important is the workplace climate, which needs to be in convergence with the personal level values. Organizations may adopt fair and reasonable practices to nurture a favorable environment to avoid value conflicts. For instance, the Indian university system expects every teacher to indulge in teaching, research, consultancy, and administrative work without examine one’s interest. There is an urgent need to adopt a tenure track system in the Indian context to align individual interests with organizational requirements.

## Limitations, Scope for Future Studies, and Conclusion

The limitations of the study are as follows. Firstly, the present study is based on overall gratitude, PsyCap, and spiritual climate scores. It would be interesting to explore the role of different dimensions of gratitude (sense of abundance, simple appreciation, and appreciation for others) in ensuring workplace happiness. Similarly, the moderating effect of various constructs of spiritual climate (swadharma, loksangraha, authenticity, and sense of community) may also provide some insights to future researchers. Secondly, the study is limited to the Delhi-NCR region, India. It might create an issue with the generalization of the result. Future research could collect data from teachers across the country to test the broader application of the findings. And lastly, the study is confined to the workplace happiness of teachers only. Future research could replicate the study with employees of different industries and sectors.

## Data Availability Statement

The datasets presented in this article are not readily available because data could not be shared due to promise of confidentiality and no-sharing with the respondents. Requests to access the datasets should be directed to corresponding author.

## Ethics Statement

Ethical approval was not provided for this study on human participants because the study did not experimented rather collected data through a questionnaire. Respondents were informed of the objectives of the study and thereafter informed consent was taken from them. The patients/participants provided their written informed consent to participate in this study.

## Author Contributions

NG and MM conceptualized the study and collected the data. SP and JB contributed significantly in writing and proofreading of the article. All authors contributed to the article and approved the submitted version.

## Conflict of Interest

The authors declare that the research was conducted in the absence of any commercial or financial relationships that could be construed as a potential conflict of interest.

## Publisher’s Note

All claims expressed in this article are solely those of the authors and do not necessarily represent those of their affiliated organizations, or those of the publisher, the editors and the reviewers. Any product that may be evaluated in this article, or claim that may be made by its manufacturer, is not guaranteed or endorsed by the publisher.
